# The burden of chronic respiratory diseases in adults in Nepal: A systematic review

**DOI:** 10.1177/1479973121994572

**Published:** 2021-07-06

**Authors:** Winifred Ekezie, Alex Robert Jenkins, Ian Philip Hall, Catrin Evans, Rajendra Koju, Om Prakash Kurmi, Charlotte Emma Bolton

**Affiliations:** 1 NIHR Nottingham BRC Respiratory Theme, School of Medicine, University of Nottingham, Nottingham, UK; 2 Division of Epidemiology, School of Medicine, University of Nottingham, Nottingham, UK; 3 Nottingham Centre for Evidence Based Healthcare, School of Health Sciences, 6123 University of Nottingham, Nottingham, UK; 4 Department of Medicine, 375889 Dhulikhel Hospital, Dhulikhel, Nepal; 5 Department of Medicine, 3710 McMaster University, Hamilton, Ontario, Canada

**Keywords:** Chronic respiratory disease, lung function, prevalence, Nepal, global lung health

## Abstract

While chronic lung disease causes substantial global morbidity and mortality, global estimates have primarily been based on broad assumptions. Specific country data from low-income countries such as Nepal are limited. This review assessed primary evidence on chronic respiratory disease burden among adults in Nepal. A systematic search was performed in June 2019 (updated May 2020) for studies through nine databases. High levels of heterogeneity deemed a narrative synthesis appropriate. Among 27 eligible studies identified, most were low-moderate quality with cross-sectional and retrospective study design. Chronic lung diseases identified were chronic obstructive pulmonary disease (COPD), asthma, bronchiectasis and restrictive lung diseases. Studies were categorised as: (i) community-based, (ii) hospital-based and (iii) comorbidity-related and disease burden. Reported disease prevalence varied widely (COPD, 1.67–14.3%; asthma, 4.2–8.9%). The prevalence of airflow obstruction was higher among rural dwellers (15.8%) and those exposed to household air pollution from domestic biomass burning as opposed to liquid petroleum gas users (Odds Ratio: 2.06). Several comorbidities, including hypertension and diabetes mellitus added to the disease burden. The review shows limited literature on lung disease burden in Nepal. Publications varied in terms of overall quality. Good quality research studies with prospective cohorts related to respiratory conditions are required.

## Introduction

The burden of chronic lung disease worldwide is huge, both in terms of morbidity and mortality. It is increasing and the current COVID-19 pandemic is likely to add further. Just one lung non-communicable disease (NCD), chronic obstructive pulmonary disease (COPD), causes >3 million deaths per year globally, >90% of these occurring in low to middle-income countries (LMICs).^1,2^ Globally identified risk factors in LMICs include tobacco smoke, household and ambient air pollution, and occupational exposure.^3–8^ The risk factors for developing chronic lung diseases will vary from country to country. The Himalayas contain several LMICs, many of which share geographical and cultural features. We chose Nepal as an exemplar Himalayan LMIC, with a population of circa 30 million and the 16th poorest country worldwide. Figures on disease prevalence for Nepal have been reported as part of the Global Burden of Disease project, these are primarily based on modelled data and are therefore dependent upon broad assumptions that may lack accuracy.^9^ Reportedly, NCDs in Nepal account for 66% of mortality, of which chronic respiratory diseases are ranked as the second highest cause of death (10%), alongside cardiovascular diseases (30%) and cancer (9%).^10^ The gravity of chronic respiratory diseases in Nepal was highlighted recently, with estimates second worse only to Kazakhstan for global mortality (Kazakhstan: 114.28 versus 100.75 deaths in Nepal per 100,000).^11,12^

Respiratory disease greatly impacts health service demand in Nepal through hospitalisations.^13–15^ According to the 2016–2017 Department of Health services in Nepal, bronchial asthma and COPD were the second and third most common causes of outpatient department morbidity, with cardiovascular conditions as the leading cause.^12^ Many patients reported having more than one long-term health condition.

Within Nepal, risk factors for chronic respiratory diseases vary according to gender and geographical location. Over 80% of the population lives in rural settings where a large proportion uses biomass as a source for heating and cooking fuel within poorly ventilated living quarters, with exposure to high levels of household air pollution.^16,17^ As women generally do the cooking, this disproportionately affects them. Smoking is common, particularly in men (27% vs 6% in women) and in urban areas (30% of households); in addition, there is significant ambient, agricultural, and industrial pollution.^17–20^ These exposures do not only affect respiratory disease but also contribute to other health complications and socioeconomic burdens.^6,21^ The remoteness of some rural locations hinders alternative energy sources and restricts fuel choices. Other socio-demographic factors, including illiteracy, poverty, distance and remote access to healthcare facilities, also have an adverse impact on health.^8,22^

The government of Nepal has begun taking action with policies including the development of the Integrated NCDs Prevention and Control Policy of Nepal and the Multisectoral Action Plan for the Prevention and Control of Non-Communicable Diseases (2014–2020).^23^ However, innovative health systemic structures and a comprehensive understanding of the burden of diseases are required for effective implementation.^24^ This systematic review aims to synthesise the current available evidence across community and hospital settings on the prevalence of chronic respiratory disease (excluding cancer), respiratory symptoms, lung function and lung-related burden in Nepal.

## Methods

### Search strategy

Initial scoping in May 2019 through MEDLINE, Embase, Cochrane library and manually, identified no existing systematic reviews on this area.

The protocol was registered with PROSPERO (CRD42019138552).^25^ Database searches were conducted in June 2019 and updated in May 2020 for publications, with no date restrictions. Nine electronic databases were searched: Ovid (MEDLINE, Embase, Global Health, CAB Abstracts), CINAHL, Scopus, Web of Science, Cochrane Library and Nepal Journal Online (NEPJOL). Grey literature searches were conducted, including Nepal Government/Health Ministry reports, World Health Organisation (WHO) data and OpenGrey. Searches were supplemented by contact with study authors where additional information was required as well as forward and backward citation tracking from included studies. A sample search strategy is shown, Appendix 1.

### Study screening and selection

Study designs reporting quantitative population-level prevalence or incidence of chronic respiratory disease, lung function, respiratory symptoms or burden of lung disease in adults (individuals aged 16 years and older) living in Nepal were considered. The review included only peer reviewed publications, but grey literature set the scene in the introduction. Language was not an exclusion criterion. For this review, publications studying specific occupational exposure were excluded. Conference abstracts were excluded following screening for full-text peer-reviewed publications.

**Figure 1.**
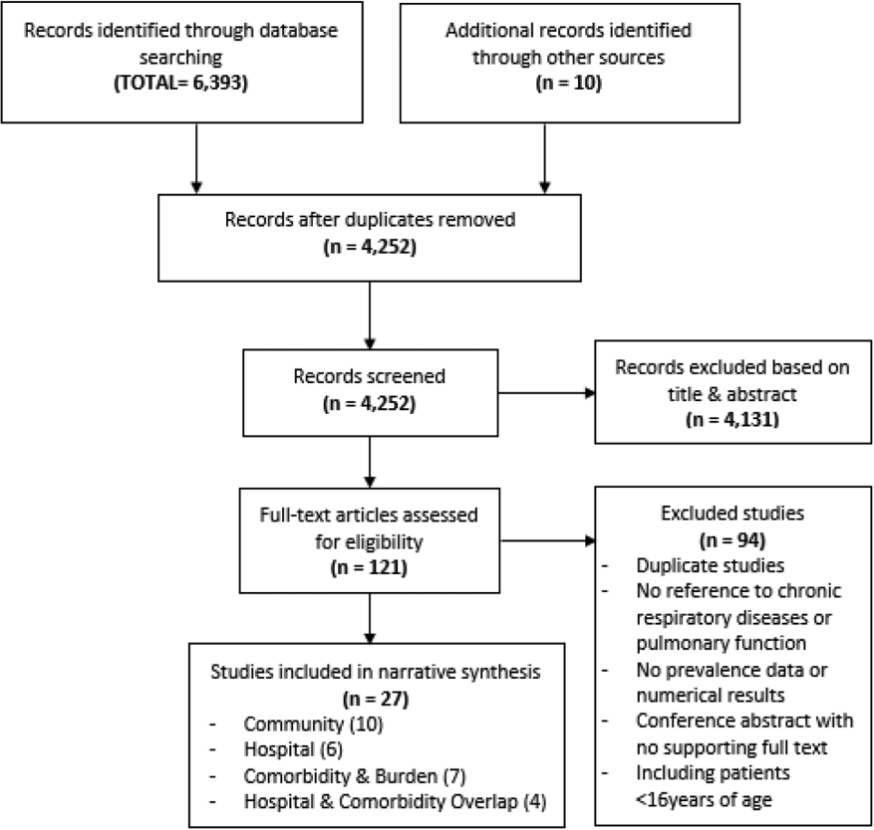
PRISMA flow chart of included studies.

Study records identified were exported into Mendeley (London, UK), with duplicates removed. Titles and abstracts were screened by two independent reviewers (WE, CEB). Full texts of potentially eligible papers were retrieved and assessed independently by two reviewers with discrepancies resolved through discussion. The Preferred Reporting Items for Systematic Reviews and Meta-Analyses (PRISMA)^26^ flow diagram of the search and screening process is shown in Figure 1.

### Data extraction and quality assessment

A standardised data extraction form was used for the final selection of studies. Data were extracted by one reviewer and checked for accuracy by a second reviewer. Quality of the included studies was independently assessed by two researchers from WE, ARJ and CEB using the AXIS critical appraisal checklist for cross-sectional studies.^27^ In keeping with the AXIS method, the quality is not judged as a numerical scoring of the component parts and therefore has a subjective element.

### Data synthesis

Studies were highly heterogeneous with huge variation used for data acquisition, analysis and reporting. Therefore the pre-planned meta-analysis was not possible (see PROSPERO: CRD42019138552), and the findings were synthesised narratively.^28^ Early on, it became apparent that the review would be in three distinct sections, given the heterogeneity of sites in the manuscripts: i) community setting, ii) hospital (mostly inpatients) setting and then iii) comorbidity-related and lung-related burden of disease.

## Results

### Study selection

A total of 6,393 papers were identified. Followng removal of duplicates, 4,252 studies were screened by titles and abstracts, and 121 full-text studies reviewed (Figure 1), with 27 articles included (Tables 1 [Table table2-1479973121994572]–3).

**Table 1.** Community-related chronic respiratory disease prevalence studies.

Author	Study design	Diseases and Symptoms	Study setting	Exposures	Disease outcomes	Respiratory and Symptoms	Lung function outcomes	Quality Range
Acharya et al. ^37^	Cross-sectional survey	Asthma	Community older adults (≥60 years) Sample: 401 participants *(Self-report)*	Not specified	**Asthma**: 25.4%	Not reported	Not conducted	Moderate
Dhimal et al.^50^	Cross-sectional	COPD	Nationwide community survey of adults >20 years Sample: 12,557 (prevalence determined in 11,277) participants (Self-report)	Not specified	**COPD prevalence** (95% CI): 11.7% (10.5–12.9) Increasing prevalence with increasing age range: 20–39 yrs: 6.7% (4.9–7.7) 40–59 yrs = 10.5% (9.1–12.1) >60 yrs = 21.5% (19.3–24.1) Male/female = 12.6% (11.2–14.1) vs 11.0% (9.6–12.4) Rural/urban = 11.7% (10.0–13.6) vs 11.7% (10.1–13.4)	Not assessed	Not conducted	Low
Joshi et al.^32^	Cross-sectional	COPD Asthma symptoms Lung function	Community rural adult kitchen dwellers Sample: 154 participants *(Self-report)*	- Indoor Air Pollution - Smoking (never = NS, current = CS, previous = PS)	- **COPD**: 1.67% - **Bronchial asthma**: 5.58%	**Cough (25.69%), phlegm (15.08%), breathlessness (15.08%), wheezing (11.73%) Disease prevalence based on exposure duration** (hours) - **Cough**: <2 (8, 5.19%), 2 to <4 (27, 17.53%), ≥4 (11, 7.14%), (p < 0.05) - **Phlegm**: <2 (1, 0.65%), 2 to <4 (15, 9.74%), ≥4 (11, 7.14%), (p < 0.05) - **Breathlessness**: <2 (0, 0%), 2 to <4 (15, 9.74%), ≥4 (11, 7.14%), (p < 0.05) - **Wheezing**: <2 (0, 0%), 2 to <4 (10, 6.49%), ≥4 (11, 7.14%), (p < 0.05) - **COPD**: <2 (0, 0%), 2 to <4 (2, 1.30%), ≥4 (1, 0.65%), (p < 0.05) - **Bronchial asthma**: <2 (0, 0%), 2 to <4 (5, 3.25%), ≥4 (5, 3.25%), (p < 0.05) **Diseases Symptoms** - **Cough (n = 46):** NS (25.5%), CS (26.44%), PS (18.18%) (p < 0.01) - **Phlegm (n = 27):** NS (10.63%), CS (16.52%), PS (18.18%) (p < 0.01) - **Breathlessness (n = 27):** NS (10.63%), CS (15.70%), PS (27.27%) (p < 0.01) - **Wheezing ( n = 21 )**: NS (10.63%), CS (11.57%), PS (18.18%) (p < 0.01)	PEFR conducted but % predicted values not used	Low
Kurmi et al.^47^	Cross-sectional	Lung function	Community rural and urban population 16 years and above Sample: 1,648 participants *1,392 (valid spirometry)*	**Rural:** Household Air Pollution (Biomass) **Urban**: Outdoor Air Pollution	Not assessed	Not reported	Airflow obstruction comparison of biomass fuel to liquefied petroleum use: OR = 2.06 (1.16–3.67), p = 0.014	High
Kurmi et al.^48^	Cross-sectional	symptoms Lung function	Community rural and urban population 16 years and above Sample: 1,648 participants *(Self-report)*	**Rural:** Household Air Pollution (Biomass) **Urban**: Outdoor Air Pollution	Not assessed	Male (Rural, n = 382 vs Urban, n = 380), Female (Rural, n = 463 vs Urban, n = 423) **- Wheeze ever:** male (23.5% vs 8.7%, p < 0.001), female (32% vs 10.3%, p < 0.001) **- Chronic cough:** male (4.7% vs 5.3%, p = 0.719), female (4.4% vs 4.2%, p = 0.864) **- Chronic phlegm:** male (3% vs 12.9%, p < 0.001), female (4% vs 5.8%, p = 0.223) **- Dyspnoea**: male (12% vs 2.5%, p < 0.001), female 17.8% vs 7.6%, p < 0.001)	**Airflow obstruction after exposure to Biomass** (OR (95% CI, p-value) **FEV1/FVC < 0.70:** (male 1.94 (1.05– 3.59, p = 0.035) vs female 1.30 (0.67 –2.54, p = 0.436) **FEV1/FVC < LLN:** (male 1.11 (0.41–3.00, p = 0.84) vs female 1.67 (0.66– 4.23, p = 0.281)	High
Pandey et al.^29^	House to house survey	Chronic bronchitis Lung function	Community rural adults in hill region 20 years and above Sample: 2,826 participants	Not specified	Crude prevalence ( n = 2,826): Chronic bronchitis * (18.3%), Emphysema (3.1%), Cor pulmonale (1.5%) * Chronic bronchitis defined using the MRC definition	Not reported	Spirometry in a representative sample of chronic bronchitis (n = 61), 35 (57.4%) had airflow obstruction, Spirometry in a representative sample of 146, 23 (15.8%) had airflow obstruction.	Moderate
Prasad et al.^33^	Cross-sectional	Lung function	Community women, 30–40 years old Sample: 200 participants *(Self-report)*	Smoking	Not assessed	Not reported	**Lung function test (mean ± SE)** **FVC (ml):** Smokers (2700 ± 0.045), Non-smokers (3400 ± 0.07) **FEV (ml):** Smokers (2310 ± 0.03), Non-smokers (2727 ± 0.02) **PEFR (l/min):** Smokers (280 ± 0.09), Non-smokers (332 ± 0.06)	Low
Pratali et al.^38^	Cross-sectional	COPD Lung function	Community rural village residents 16–75 years Sample: 32 houses (78 villagers) *(Lung function test)*	Biomass fuels	**Bronchial obstruction:** 6/78 (7.7%) of which 5/78 had a **non-reversible bronchial obstruction**, classed as COPD 6%	Not reported	**FEF_25_ _–_ _75_ :** <80% predicted in 54%	Moderate
Ranabhat et al.^20^	Cross-sectional	Asthma Symptoms	Community rural households Sample: 157 houses *(Self-report)*	Indoor Air Pollution	Asthma: 8.9%	Reported IAP related health problems based on multiple responses (n = 297) Includes Difficulty in breathing (27.3%), Productive cough (19.7%), Dry Cough (8.9%), Tearing of eyes (55.4%)	Not conducted	Moderate
Shrestha and Shrestha^36^	Cross-sectional	COPD Asthma Symptoms	Community rural and urban households Sample: 98 houses (168 villagers) (Rural, n = 140 vs Urban, n = 28)	Indoor Air Pollution *(Processed, n = 43 vs Unprocessed fuel, n = 125)*	**Prevalence (Processed vs Unprocessed fuel); (Rural vs Urban)** **- COPD:** 14.3% (7% vs 16.8%); (16.4% vs 3.6%) **- Asthma:** 4.2% (0% vs 5.6%); (4.3% vs 3.7%)	Prevalence (Processed vs Unprocessed fuel); (Rural vs Urban) **Cough:** 31.5% (14% vs 37.6%); (35% vs 14.3%) **Phlegm:** 20.2% (9.3% vs 24%); (22.9% vs 7.1%) **Cough and Phlegm**: 15.5% (7% vs 18.4%); (17.1% vs 7.1%) **Breathlessness**: 27.4% (11.6% vs 32.8%); (30% vs 14.3%) **Wheezing:** 23.2% (7% vs 28.8%); (26.4% vs 7.1%) **Breathlessness and wheezing:** 20.2% (7% vs 24.8%); (22.9% vs 7.1%) **At least one respiratory symptom:** 35.7% (14% vs 43.2%); (40% vs 14.3%) **All respiratory symptoms:** 11.9% (4.7% vs 14.4%); (15.7% vs 3.7%)	Not conducted	Low

Airway Obstruction (AO), Confidence Interval (CI), Chronic Obstructive Pulmonary Disease (COPD), Indoor Air Pollution (IAP), Lower Limit of Normal (LLN), Peak Expiratory Flow Rate (PEFR), Forced Expiratory Volume in one second (FEV_1__)_, Forced Vital Capacity (FVC), Forced Expiratory Flow at 25–75% of FVC (FEF, Standard Error (SE)).

**Table 2. table2-1479973121994572:** Hospital-related chronic respiratory disease studies.

Author	Study design	Disease /state	Study setting	Disease outcomes	Quality Range
Amundsen et al.^42^	Retrospective chart-review	- COPD	Hospitalised patients Sample: 1,139 patients adult non-obstetric, non-traumatic hospitalised cases (Clinical diagnosis) *Duration: 4 months*	**Non-communicable diseases:** 332 of the 1139 of which 45% (n = 148) were **COPD** COPD accounted for 13% of all adult, non-obstetric, non-traumatic admissions Of the 148 with COPD: **- Gender**: male (40%), female (60%) **- Location**: urban (51%), village (49%)	Moderate
Bhandari and Sharma^44^	Descriptive cross-sectional study	- COPD	Hospitalised COPD patients Sample: 313 patients (Clinical diagnosis) *Duration: 3 years*	Of the 313 with COPD: **- Gender:** male (40%), female (60%) Patients presenting more in cold months, if reside closer to hospital and higher ethnic class	Low
Bhandari et al.^43^	Cross-sectional study	- COPD	Hospitalised patients, 35 years and above across 28 non-specialised institutions Sample: 10,635 cases randomly selected (Clinical diagnosis) *Duration: 8 months*	**NCDs** accounted for 31% (n = 3294). Of the 3294 in non-specialised hospitals, COPD accounted for 43% of the NCD admissions Therefore, 14% of 10635 admissions.	Moderate
Dhungel et al.^31^	Cross-sectional	- COPD	Hospitalised patients on a medical ward Sample: 1,366 patients (Clinical diagnosis) *Duration: 2 months*	COPD: 17.3%	Low
Ghosh et al.^45^	Descriptive cross-sectional study	- COPD	Hospitalised patients of all ages to one hospital Sample: 62,446 inpatients records (Clinical diagnosis) *Duration: 3 years*	**COPD:** 2.6%	Low
Ghimire et al.^51^	Prospective observational study	- ILD	Hospitalised patients in the department Sample: 10,894 patients (Clinical diagnosis) *Duration: 2 years*	**ILD:** 44 (0.4%) <40 years = 4 (9.1%), 40–59 years =10 (22.7%), 60–69 years = 13 (29.5%), >70 years = 17 (38.6%) Of the patients with ILD, 80% were IPF	Low
Giri et al.^53^	Prospective observational study	- COPD - Asthma	>16 year-olds presenting to the emergency department at one hospital Sample: 21,892 patients (Clinical diagnosis) *Duration: 3 years*	Patients with known presenting complaint, n = 21,892; respiratory complaints, n = 2,018 (9.2%); short of breath, n = 1,572 (7.2%); COPD or asthma, n = 106 (0.5%); other respiratory, n = 340 (1.6%)	Moderate
Prajapati and Pradhan^49^	Descriptive cross-sectional study	- COPD - Bronchial asthma - Restrictive lung disease	Outpatients referred for spirometry to a hospital clinic Sample: 755 patients (Clinical diagnosis) *Duration: 16 months*	Diagnoses made from spirometry: COPD (31.4%), Asthma (24.2%), Restrictive lung disease (8.1%) Association between smoking history and abnormal spirometry (smokers vs non- smokers); OR (% CI), p-value **COPD**: (78.5% vs 21.5%); 10.43 (6.91–15.73), p < 0.0001 **Asthma**: (43.2% vs 56.8%); 2.17 (1.46–3.23), p < 0.0001 **Restrictive disease:** (47.5% vs 52.5%); 2.59 (1.46–4.58), p < 0.001	Moderate
Shankar et al.^34^	Retrospective case notes review observational study	- COPD	Hospitalised patients across several departments, aged ≥60 years to one hospital Sample: 548 patients (Clinical diagnosis) *Duration: 1 year*	Data not interpretable (Conflicting reporting between results and discussion sections in relation to hospital admissions for AECOPD)	Low
Shrestha et al.^54^	Descriptive cross-sectional study	- COPD - Asthma	>18-year-old patients attending the emergency department at one hospital Sample: 1,200 patients *Duration: 14 months*	107 admissions presenting with dyspnoea (9% total) History of COPD among the 107 dyspnoea admissions, n = 68 (63.3%) Asthma among dyspnoea admissions, n = 3 (2.8%) Emergency department diagnosis of 107 dyspnoea complaints: Respiratory, n = 56 (52.3%) Respiratory and cardiovascular, n = 14 (13.1%) Metabolic, n = 12 (11.2%) Respiratory with others. n = 10 (9.3%) Neuropsychiatric, n = 7 (6.7%) Cardiovascular, n = 4 (3.7%) Gastrointestinal, n = 4 (3.7%)	Low

Acute Exacerbation of Chronic Obstructive Pulmonary Disease (AECOPD), Chronic Obstructive Pulmonary Disease (COPD), Non-Communicable Disease (NCD), Interstitial Lung Disease (ILD), Idiopathic Pulmonary Fibrosis (IPF).

**Table 3.** Comorbidity-related and burden of chronic respiratory disease studies.

Author	Study design	Disease	Study setting	Co-morbid disease outcomes	Symptoms and/ or other outcome associations	Quality Range
Bhatta et al.^30^	Retrospective observation	Bronchiectasis	Symptoms and co-morbid presence of pulmonary hypertension and cor pulmonale in patients with bronchiectasis (outpatients) *Sample: 100 patients*	Pulmonary arterial hypertension and cor pulmonale (50%)	Cough (100%), haemoptysis (75%), worsening shortness of breath (70%), fever (50%), abnormal chest physical examination (75%)	Moderate
Dhungel et al.^31^	Cross-sectional	COPD	Co-morbid presence of cardiovascular conditions in patients with COPD (inpatients) *Sample: 237 COPD patients*	Hypertension (41.3%), diabetes (5%), coronary artery disease (5.9%), dilated cardiomyopathy (4%)	Not reported	Low
Ghosh et al.^45^	Descriptive cross-sectional study	COPD	Mortality rate of patients admitted with COPD *Sample: 62,446 inpatients records of which 1604 had COPD*	Not assessed	Inpatient mortality 6% from the 1604 patients admitted with COPD	Low
Ghimire et al.^51^	Prospective observational study	ILD	Co-morbid conditions, symptoms and survival rate in patients with ILD *Sample: 10,894 patients of which 44 had ILD*	Gastro-oesophageal reflux disease = 20 (45.4%), Diabetes mellitus = 7 (15.9%), Hypertension = 7 (15.9%), Cor pulmonale = 6 (13.6%), Renal impairment = 5 (11.4%), IHD = 4 (9.1%), OSA = 2 (4.5%), Pulmonary tuberculosis = 2 (4.6%), Hypothyroidism = 1 (2.3%)	Symptoms: Fever = 15 (34.1%), Cough = 43 (97.7%), Dyspnoea = 42 (95.5%), Sputum = 6 (13.6%), Chest pain = 13 (29.5%), Orthopnoea =4 (9.1%), Haemoptysis = 2 (4.5%) Survival rate - 5 months = 0.95 ± 0.03, 12 months = 0.84 ± 0.1, 24 months = 0.42 ± 0.2	Low
Giri et al.^53^	Prospective observational study	COPD Asthma	Mortality rate in patients presenting to the emergency department with a diagnosis of COPD or asthma *Sample: 21,892 patients*	Not assessed	Any presenting complaint and a diagnosis of asthma or COPD had a 90-day mortality rate of 32%	Moderate
Koirala et al.^52^	Cross-sectional study	COPD Asthma ILD Bronchiectasis	Comorbidities and characteristics of hospital outpatients diagnosed with chronic respiratory disease Sample: 50 patients (COPD = 24; asthma = 18; bronchiectasis = 6; ILD = 2)	**Sleep-related breathing disorders in patients with chronic respiratory disease** COPD = 15 (62.5%) Asthma = 10 (55.6%) Bronchiectasis = 3 (50%) ILD = 1 (50%) Systemic hypertension – 36%; diabetes mellitus – 16%, metabolic syndrome – 24%, heart failure – 6%, IHD – 6%.	Not reported	Moderate
Hirachan et al.^46^	Cross-sectional	COPD	Evaluation of the right heart of patients with COPD attending echocardiography clinic (inpatients) *Sample: 50 COPD patients*	All patients had pulmonary hypertension, 60% were severe, 28% were moderate, and 12% were mild	Significant ECG abnormality (94%), P pulmonale (90%), Atrial arrhythmias/fibrillation or Multifocal tachycardia (12%)	Moderate
Shrestha et al.^35^	Prospective observation	COPD	Co-morbid presence of cardiovascular conditions in patients with COPD (inpatients) *Sample: 507 patients (mainly Brahmin (52.7%))*	Selected respiratory and cardiovascular comorbidities (n = 501) Chronic cor pulmonale (27.3%) Type II respiratory failure (10.4%), Hypertension (9.9%), dilated cardiomyopathy (2.4%), valvular heart disease (1.5%), IHD (1.6%), congestive cardiac failure (3.1%), left ventricular failure (1.8%), Other cardiovascular diseases (3.3%), diabetes mellitus (3.7%)	Not reported	Low
Sijapati et al.^39^	Prospective observation	COPD	Factors determining outcomes in hospitalised AECOPD Sample: 100 patients *COPD stage I (8%), stage II (61%), stage III (31%)*	Hypertension (15%), diabetes mellitus (4%) and IHD (10%)	- **Deaths**: 20 of the 100	Low
Sijapati et al.^40^	Hospital-based prospective observation	COPD Bronchiectasis	Co-morbid presence of bronchiectasis in patients presenting with COPD (both out- and inpatients), without a former bronchiectasis history. CT done to confirm bronchiectasis. *Sample: 120 patients*	**COPD patients (n= 120):** With bronchiectasis (COPD-B) n = 53 (44%), without bronchiectasis (COPD-nB) n = 67 (56%)	**COPD-B (n = 53), COPD-nB (n = 67) Patients with COPD plus bronchiectasis:** Had less daily breathlessness More had purulent sputum More had had an exacerbation requiring admission in last year.	Low
Thapa et al.^41^	Cross-sectional	COPD	Co-morbid presence of anxiety and depression in patients with COPD patients compared to the general population (Outpatients) *Sample: 198 patients*	**Beck anxiety and depression inventory scale scores** (mean ± SD) - **COPD group:** anxiety (23.76 ± 9.51), depression (27.72 ± 9.37) - **General population:** anxiety (8.01 ± 6.83), depression (11.60 ± 8.42) Both greater in COPD compared to general (p < 0.001)	Not reported	Moderate

Acute Exacerbation of Chronic Obstructive Pulmonary Disease (AECOPD), Standard Deviation (SD), Obstructive Sleep Apnoea (OSA), Ischaemic Heart Disease (IHD), Interstitial Lung Disease (ILD), Electrocardiogram (ECG), Computed tomography (CT).

### Overview of included studies

Years of publication ranged from 1984 to 2019, and all were published in English. One was published in the 1980s,^29^ 7 between 2000 and 2010,^30–36^ and 19 after 2011.^20,37–54^ All included studies were cross-sectional, direct observations of clinical notes, comparative and single point survey designs.^20,29–54^ The studies reported on diseases (usually self-reported), symptoms, related health conditions and lung function. Most of the studies were either of low (n = 13) or moderate (n = 12) quality, with two classed as high quality.

Some studies reported on more than one condition, with the major diseases identified being: COPD (22 studies),^31,32,34–36,38–54^ asthma (8 studies),^20,32,36,37,49,52–54^ bronchiectasis (2 studies and as a co-morbidity in one),^30,40,52^ restrictive lung diseases (3 studies),^49,51,52^ and ‘chronic bronchitis’ (1 study).^29^

Symptoms reported included chronic cough, phlegm or sputum production, breathlessness or shortness of breath, chest tightness, wheezing, dyspnoea and sore throat. Reporting included presence of pulmonary arterial hypertension^30^ and cor pulmonale.^29,30,35,51,52^

Lung function was reported in seven studies,^29,32,33,38,47,48,51^ and data presented across different spirometric measures, with one reporting proportions only of patients categorised as ‘normal’, ‘obstruction’ and ‘restriction’.^51^

### Community-based prevalence studies

Studies included over 18,000 participants across diverse population groups (Table 1). COPD and asthma, (usually self-reported), were covered across small-medium sized studies of 78 to 401 participants,^20,32,36–38^ with one large-scale study of 12,557 participants (prevalence determined in 11,277).^50^ A study of chronic bronchitis, according to the Medical Research Council definition, was based on 2,826 participants.^29^

One study on both rural and urban households reported a COPD prevalence of 14.3%.^36^ This was comparable with the findings of Dhimal et al.,^50^ which reported a nationwide prevalence of COPD of 11.7%. These findings differed from other studies, where reported COPD prevalence was 1.67% in rural adult kitchen dwellers (reported in the paper as adults who spend a large amount of the day in the kitchen),^32^ and 6% COPD prevalence in rural community residents using biomass fuels.^38^ Meanwhile, prevalence of chronic bronchitis of 18.3% was reported in a study with an additional 3.1% with emphysema too.^29^ Asthma prevalence ranged from 4.2% to 8.9%^20,32,36^ but one study reported 25.4% in adults ≥60 years.^37^ Generally, chronic respiratory diseases were more prevalent in adults aged over 40 years.^37,38,50^

In a general representative population, symptom-wise, one study reported cough, phlegm, breathlessness and wheezing to each have a prevalence of over 20%, and greater among populations using unprocessed fuels and living in rural regions, than in those using processed fuels and living in urban households.^36^ This agreed with Ranabhat et al.,^20^ where reported breathlessness and productive cough were 27.3% and 19.7% of individuals living in rural communities exposed to household air pollution. Kurmi et al.^47^ reported that wheeze (male, 23.5% vs 8.7%; female, 32% vs 10.3%) and dyspnoea (male, 12% vs 2.5%; female, 17.8% vs 7.6%) were more prevalent symptoms in the rural environment as a whole, whereas chronic phlegm (male, 4.7% vs 5.3%; female, 4.4% vs 4.2%) and cough (male, 3% vs 12.9%; female, 4% vs 5.8%) were more comparable between rural and urban environments.^48^ Symptom reporting was also greater in current and past smokers than never smokers, with the prevalence of cough, phlegm, breathlessness and wheezing all above 11% in rural community kitchen dwellers as a whole.^32^

Kurmi et al.,^47,48^ a large study, reported across two publications that the odds ratio of having airflow obstruction with exposure to biomass was two times higher (OR: 2.06)^47,48^ compared to liquid petroleum gas users. Males (OR: 1.94; p = 0.035) were more at risk of suffering airflow obstruction after exposure to biomass (OR: 1.30; p = 0.436) when the threshold of FEV_1_/FVC < 0.70 was utilised but not for a threshold of FEV_1_/FVC < lower limit of normal (Males, OR: 1.11, p = 0.840; Females, OR: 1.67, p = 0.281). Airflow obstruction was reported in 15.8% of rural community residents using a subset representative of the overall study population, with airflow obstruction prevalent in 57.4% among those meeting the study criteria for chronic bronchitis.^29^ Smokers had lower lung function in one study compared to those who reported to not smoke, although it was unclear whether this comparator group were ex- or never smokers^33^. Pratali et al.^38^ found that 54% of rural community residents using biomass fuels had a FEF_25__–__75_ < 80% predicted. Joshi et al.^32^ reported absolute peak flow and not % predicted, and therefore is largely uninterpretable.

### Hospital-based studies

Ten studies, including a total of 111,188 subjects reported on the prevalence of chronic respiratory diseases in a hospital setting (Table 2).^31,34,42–45,49,51,53,54^ The majority of studies reported on COPD among hospitalised patients,^31,34,42–45^ with other studies reporting on interstitial lung disease (ILD),^51^ both COPD and asthma,^53,54^ and outpatient spirometry referrals.^49^

Overall estimates for the proportion of admissions due to COPD varied widely from 2.6% to 14% in studies including all hospital admissions and up to 17.3% in medical wards.^31,42,43,45^ Two studies reported that among hospitalised participants with an NCD, 43–45% were COPD patients.^42,43^ In two studies, the majority of hospitalised COPD patients were females (60%).^42,44^

One study reporting on dyspnoea admissions (this being 9% of all the admissions) at one hospital, identified that 52.3% received a respiratory diagnosis for that presentation, another 13% had a combined respiratory and cardiovascular cause, and 9.3% received a diagnosis of respiratory accompanied with other complications.^54^ However, dyspnoea was also due to metabolic (11.2%), neuropsychiatric (6.7%) and cardiovascular alone (3.7%).^54^ In a study where the majority of hospital admissions were attributed to injuries in young adults, respiratory complaints still accounted for 9.2% of total hospital admissions, with COPD or asthma accounting for 0.5% of total hospital admissions.^53^

Ghimire et al.^51^ reported a prevalence of 0.4% for ILD among patients treated in hospital for pulmonary disorders. The majority of these ILD’s were attributed to idiopathic pulmonary fibrosis (79.5%), and 68.1% of the patients were over the age of 60 years.^51^

There was only one hospital-based study with spirometry for outpatients: the final diagnoses being bronchial asthma (24.2%), COPD (31.4%), and restrictive lung disease (8.1%) of those referred for testing.^49^

### Comorbidity-related and burden of disease

Eleven studies, with a total of 96,594 patients reported on comorbidity and burden of chronic respiratory disease.^30,31,35,39–41,45,46,51–53^ The major chronic respiratory diseases were COPD (seven studies),^31,35,39–41,45,46^ bronchiectasis (one study),^30^ ILD (one study),^51^ COPD or asthma (one study),^53^ or they assessed COPD, asthma, bronchiectasis and ILD together (one study) (Table 3).^52^ Reported comorbidities covered other respiratory conditions, endocrine, cardiovascular disease, sleep and mental health disorders.

Common comorbidities reported in patients with COPD were hypertension ranging 9.9–41.3% of subjects,^31,35,39^ diabetes mellitus: 3.7–5%,^31,35,39^ ischaemic heart disease/coronary artery disease: 1.6–10%,^31,35,39^ dilated cardiomyopathy: 2.4–4%,^31,35^ chronic cor pulmonale: 27.3%,^35^ and type II respiratory failure: 10.4%.^35^ One study in patients with COPD who had echocardiography reported that all had echocardiography evidence of pulmonary hypertension, of which 60% were severe.^46^ Another study reported a co-morbid presence of bronchiectasis in 44% of COPD patients.^40^ Anxiety and depression were almost three times more common in COPD patients compared to the general population.^41^

One study reported that patients with an initial diagnosis of ILD had a 45.4% prevalence of gastro-oesophageal reflux disease, 15.9% diabetes mellitus, 15.9% hypertension, 13.6% cor pulmonale, 11.4% renal impairment, and 9.1% ischaemic heart disease.^51^ Systemic hypertension (36%), diabetes mellitus (16%), metabolic syndrome (24%), ischaemic heart disease (6%) and heart failure (6%) were prevalent across multiple chronic respiratory diseases (COPD, ILD, bronchiectasis and asthma).^52^ Two studies reported on sleep disorders with Ghimire et al.^51^ reporting obstructive sleep apnoea prevalence in 4.5% of ILD patients, while Koirala et al.^52^ reported that 62.5% of COPD and 55.6% of asthma patients had sleep-related breathing disorders.

Two studies reported mortality rates ranging from 6–20% in patients admitted to hospital with COPD.^39,45^ Another study reported a 90-day mortality rate of 32% for patients admitted to the hospital with any complication with an accompanying diagnosis of COPD or asthma.^53^ The prevalence of an exacerbation requiring hospitalisation was nearly double in COPD patients with coexistent bronchiectasis compared to those without (56.6% vs 26.8%).^40^ Ghimire et al.^51^ reported survival rates of 95% at 5 months, 84% 1-year, and 42% 2-year following ILD diagnosis.

In patients with bronchiectasis or ILD, common symptoms experienced included cough (bronchiectasis, 100%; ILD, 97.7%), haemoptysis (bronchiectasis, 75%; ILD, 4.5%), worsening shortness of breath (bronchiectasis, 70%; ILD, 95.5%), and fever (bronchiectasis, 50%; ILD, 34.1%).^30,51^

## Discussion

This study has identified and synthesised the available evidence on the prevalence of chronic respiratory diseases, symptoms, lung function, comorbidities and the overall burden of lung disease in Nepal. Overall, COPD is common, both in males and females, and is associated with frequent hospital admission and a range of comorbidities. Smoking and household air pollution were found to be common risk factors. Estimates of asthma prevalence varied widely and there was no good data on the ILD prevalence.

### Community-based findings

The findings highlight the high prevalence of COPD and the varied prevalence of asthma in Nepal. The global prevalence of COPD has been reported to be approximately 9.2%,^55^ with other estimates suggesting age-standardised figures for prevalence of males: 3.2% and females: 2.0%.^56^ In comparison, large-scale and high quality studies assessing COPD prevalence in representative samples of the overall population in LMIC’s such as China and India have shown prevalence to be 8.6%^57,58^ and 4.2% respectively.^59^ The prevalence estimates of 1.67% to 14.3% in Nepal vary significantly on either side of these prevalence figures, making it challenging to draw meaningful comparisons. As with COPD, asthma prevalence figures for Nepal varied widely depending on the population, rendering it difficult to interpret alongside World Health Surveys and findings from other LMICs where prevalence of diagnosed asthma have been estimated at 4.3%^60^ and 2.9% respectively.^59^ However, both asthma and COPD prevalence figures could be impacted by the misclassification of disease and by the lack of quality studies. To date, most publications reporting COPD diagnosis in Nepal are not based on spirometry, and where they are, they did not assess post-bronchodilator measurements.^61^ Similarly, the term ‘COPD’ is not universally recognised in Nepal, and therefore there is further misclassification of patients as having asthma or other terms for chronic lung disease. This review highlights that the large proportion of the prevalence studies were of moderate to low-quality evidence and utilised diverse populations and approaches.

Two publications from the same large project met the criteria for high quality.^47,48^ The use of biomass fuels as a household energy source markedly increased the risk of airflow obstruction compared to liquefied petroleum,^47^ in line with the Global Burden of Disease study and other studies.^62–65^ Further, symptoms are prevalent in both urban and rural community dwellers, but particularly in rural, where there is greater use of biomass fuels leading to household air pollution.^48^ These observations agree with previous findings.^66,67^ However, some recent evidence contradicts the lung function observations, whereby post-bronchodilator airflow obstruction was not associated with the use of biomass fuels.^68^

### Hospital-based findings

In the hospital setting, the estimates of COPD and asthma also varied across studies. The reporting of hospital admissions attributed to COPD and/or asthma in Asian LMICs is scarce in the literature providing challenges in making effective comparisons. Of the available evidence, a study looking into hospital admission causes in China reported that chronic respiratory disease contributed to 22.6% of total admissions.^69^ Dagenais et al.^70^ further suggested that respiratory disease patients accounted for around 8.5% of global hospital admissions, but the number of first admissions attributed to respiratory disease was higher (4.9%) in high income countries compared to LMIC (1.9%). It was suggested that this was due to higher diagnosis rates and earlier treatment in high income countries. ^70^

Of note, two studies highlighted that most hospitalised COPD patients were female.^42,44^ This conflicts evidence from other LMICs whereby hospital admissions for COPD appear to be more males,^71,72^ and prevalence rates of COPD are higher in males.^58,73^ This finding is of potential importance in addressing the burden of COPD in Nepal and needs to be investigated further. One possibility is the greater exposure of women to household air pollution.

It is important to note that drawing comparisons with other countries based on the low to moderate quality data are hindered by the disparities in reporting of admissions, including whether total hospital admissions, select age groups or select hospital departments and also the hospital location and access. All these factors hinder interpretation, especially when the studies tend to include large hospital centres in urban areas. This creates challenges in providing evidence relating to rural environments.

### Comorbidity-related and burden findings

A high prevalence of concurrent comorbidities included other respiratory disorders, ^35,40,51,52^ cardiovascular disease,^31,35,39,46,51,52^ diabetes,^31,35,39,51,52^ anxiety and depression.^41^ The estimates of prevalence varied markedly. Although these were small and discrete populations, the coexistent comorbidities concur with the literature in other countries and warrant further investigation and studies to delineate the additive burden, contribution to mortality, opportunity for earlier identification and prevention.^74,75^

There was minimal literature on mortality associated with chronic respiratory disease. For COPD, in-hospital mortality rates ranged from 6–20%,^39,45^ which is a wide variation in the estimate. Comparisons may be drawn with COPD in-hospital mortality rates in India, which are around 12%,^72^ but further clarity is needed to distinguish a precise estimate in Nepal. Access to hospital and possible later presentation with symptoms, healthcare costs and the provision and access of core medications and treatment once at the hospital are factors. There was no population-level COPD or other chronic lung disease mortality data for Nepal.

### Strengths and limitations

This review included self-reported and diagnosed lung disease, symptoms, lung function and comorbidity, allowing capture on the wider burden of respiratory disease. Outcomes needed to be contextualised to study setting, allowing for adequate reflection for future research and policy utilisation and a rounded view of respiratory disease burden in both the community and hospital while highlighting variation and a need for further research.

The main limitation was the considerable heterogeneity in the studies in terms of population sampling approach and data collection methods leading to a variety of outcome measures and a range of information. There were stark differences in study quality stemming from design and setting, which make it necessary to separate the study into three categories. The populations recruited to these studies should also be considered with several elements identified that infer heavy bias, potentially affecting the interpretation of results. For example, lack of segregated data based on the age of the population monitored meaning standardised results could not be produced, lack of spirometry especially including post-bronchodilator assessments to determine the overall prevalence, lack of reporting on medications, lifestyle factors, previous medical history (e.g. childhood respiratory complications), and socioeconomic status to help interpret the populations included. It is also important to note that no data on incidence of chronic respiratory diseases were available for reporting, and that reported symptom data were not consistent. As a result, the high variability required a narrative analysis approach with details of the population presented as opposed to a pooled estimate of prevalence and burden. Ultimately, these issues made it difficult to compare studies but have helped to provide an evidence base for future research needs assessing non-communicable lung disease burden in Nepal.

### Implications for future health research and care

While unable to fully delineate the estimates of the true prevalence of respiratory disease in Nepal, the scale and burden of chronic lung disease are marked. Current research is taking a population approach to systematically identify the scale of COPD in urban Nepal.^76^ Identifying the estimates and burden of disease drives the need for optimised healthcare strategies and subsequent implementation and dissemination. For example, smoking cessation to reduce tobacco exposure^77–79^ and pulmonary rehabilitation^77^ have a strong evidence base in their effectiveness in managing lung diseases. Pulmonary rehabilitation takes a holistic approach to reduce breathlessness, improve function and treat multimorbidity.^80^ Importantly, these are high value, low-cost interventions and will be of an increasing need for a country with an increasing life expectancy and a growing issue of NCD prevalence. Reducing exposures through smoking cessation and utilisation of alternative fuel sources alongside health promotion are key to the long-term management and prevention of chronic respiratory diseases in Nepal. However, any consideration and inclusion of public health interventions needs a full evaluation of their potential appropriateness and acceptability given the tremendous social, cultural, linguistic, economic and geographic diversity of Nepal. Important factors include the fact that a large proportion of people in Nepal live in very rural and often mountainous terrain, many are living in extreme poverty and illiteracy is high.^8,16,17,22^

## Conclusion

This systematic review demonstrated that there is limited published literature on the burden of lung disease and marked variation in outcome measures and populations studied. Nonetheless, chronic respiratory burden in Nepal is likely to be considerable. This is a priority area for future research. Based on the available information, there is a clear need for further studies and the need for healthcare professionals, providers and policymakers to develop effective strategies to tackle chronic respiratory diseases in Nepal. These findings are also likely to be relevant to other Himalayan LMICs.

## Appendix 1

### Search strategy from Scopus

(Nepal AND asthma) OR (Nepal AND bronchiectasis) OR (Nepal AND chronic bronchitis) OR (Nepal AND chronic obstructive pulmonary disease*) OR (Nepal AND emphysema) OR (Nepal AND idiopathic pulmonary fibrosis) OR (Nepal AND lung fibrosis) OR (Nepal AND interstitial lung disease*) OR (Nepal AND silicosis) OR (Nepal AND occupational lung disease*) OR (Nepal AND small airways disease*) OR (Nepal AND breathless*) OR (Nepal AND cough*) OR (Nepal AND wheez*) OR (Nepal AND dyspn*) OR (Nepal AND respiratory arrest*) OR (Nepal AND exacerbat*) OR (Nepal AND hypoxemia) OR (Nepal AND sputum) OR (Nepal AND phlegm) AND (LIMIT-TO (DOCTYPE,“ar”) OR LIMIT-TO (DOCTYPE,“cp”)) AND (LIMIT-TO (EXACTKEYWORD,“Human”)) AND (LIMIT-TO (SUBJAREA,“MEDI”) OR LIMIT-TO (SUBJAREA,“PHAR”) OR LIMIT-TO (SUBJAREA, “AGRI”) OR LIMIT-TO (SUBJAREA,“ENVI”) OR LIMIT-TO (SUBJAREA,“SOCI”) OR LIMIT-TO (SUBJAREA,“NURS”) OR LIMIT-TO (SUBJAREA,“HEAL”))
